# Unsupervised cluster analysis of clinical and metabolite characteristics in patients with chronic complications of T2DM: an observational study of real data

**DOI:** 10.3389/fendo.2023.1230921

**Published:** 2023-10-20

**Authors:** Cuicui Wang, Yan Li, Jun Wang, Kunjie Dong, Chenxiang Li, Guiyan Wang, Xiaohui Lin, Hui Zhao

**Affiliations:** ^1^ Department of Health Examination Center, The Second Affiliated Hospital of Dalian Medical University, Dalian, China; ^2^ Department of Gastroenterology, The 986th Hospital of Xijing Hospital, Air Force Military Medical University, Xi’an, China; ^3^ State Key Laboratory of Molecular Reaction Dynamics, Dalian Institute of Chemical Physics, Chinese Academy of Science, Dalian, China; ^4^ School of Computer Science & Technology, Dalian University of Technology, Dalian, China; ^5^ School of Information Engineering, Dalian Ocean University, Dalian, China

**Keywords:** T2DM, chronic complications, K-means, cluster analysis, metabolite

## Abstract

**Introduction:**

The aim of this study was to cluster patients with chronic complications of type 2 diabetes mellitus (T2DM) by cluster analysis in Dalian, China, and examine the variance in risk of different chronic complications and metabolic levels among the various subclusters.

**Methods:**

2267 hospitalized patients were included in the K-means cluster analysis based on 11 variables [Body Mass Index (BMI), Systolic Blood Pressure (SBP), Diastolic Blood Pressure (DBP), Glucose, Triglycerides (TG), Total Cholesterol (TC), Uric Acid (UA), microalbuminuria (mAlb), Insulin, Insulin Sensitivity Index (ISI) and Homa Insulin-Resistance (Homa-IR)]. The risk of various chronic complications of T2DM in different subclusters was analyzed by multivariate logistic regression, and the Kruskal-Wallis H test and the Nemenyi test examined the differences in metabolites among different subclusters.

**Results:**

Four subclusters were identified by clustering analysis, and each subcluster had significant features and was labeled with a different level of risk. Cluster 1 contained 1112 inpatients (49.05%), labeled as “Low-Risk”; cluster 2 included 859 (37.89%) inpatients, the label characteristics as “Medium-Low-Risk”; cluster 3 included 134 (5.91%) inpatients, labeled “Medium-Risk”; cluster 4 included 162 (7.15%) inpatients, and the label feature was “High-Risk”. Additionally, in different subclusters, the proportion of patients with multiple chronic complications was different, and the risk of the same chronic complication also had significant differences. Compared to the “Low-Risk” cluster, the other three clusters exhibit a higher risk of microangiopathy. After additional adjustment for 20 covariates, the odds ratios (ORs) and 95% confidence intervals (95%CI) of the “Medium-Low-Risk” cluster, the “Medium-Risk” cluster, and the”High-Risk” cluster are 1.369 (1.042, 1.799), 2.188 (1.496, 3.201), and 9.644 (5.851, 15.896) (all *p*<0.05). Representatively, the “High-Risk” cluster had the highest risk of DN [OR (95%CI): 11.510(7.139,18.557), (*p*<0.05)] and DR [OR (95%CI): 3.917(2.526,6.075), (*p*<0.05)] after 20 variables adjusted. Four metabolites with statistically significant distribution differences when compared with other subclusters [Threonine (Thr), Tyrosine (Tyr), Glutaryl carnitine (C5DC), and Butyryl carnitine (C4)].

**Conclusion:**

Patients with chronic complications of T2DM had significant clustering characteristics, and the risk of target organ damage in different subclusters was significantly different, as were the levels of metabolites. Which may become a new idea for the prevention and treatment of chronic complications of T2DM.

## 1 Introduction

Type 2 diabetes mellitus (T2DM) is a highly prevalent endocrine and metabolic disease worldwide (1), which causes a variety of chronic complications such as cardiovascular disease (CVD), non-alcoholic fatty liver disease (NAFLD), renal disease, diabetic neuropathies, amputation, blindness, mortality, some brain lesions and so on (2–5). T2DM and its chronic complications bring a huge burden to the global medical and economy (6, 7). Meanwhile, chronic complications of T2DM are the leading causes of CVD, blindness, end-stage renal disease, and high mortality in China (7–9). Currently, experts divide the chronic complications of T2DM into microvascular complications, atherosclerotic cardiovascular disease (ASCVD), nervous system complications (NSC), diabetic foot (DF), and others (eye, oral cavity, skin lesions, etc.) (10), to facilitate clinical diagnosis and classification of complications. Unfortunately, the current classification measure mainly based on the location of target organs of lesion damage cannot effectively reduce the occurrence of chronic complications of T2DM or slow down their progress, because this classification method fails to take into account the pathology, mechanisms, and associated risk factors of various chronic complications. Another not-so-optimistic situation is that most patients with T2DM often develop multiple chronic complications at the same time, which greatly increases the difficulty of treatment for clinicians. Therefore, we need a more accurate division way to differentiate the T2DM patients with chronic complications, it could help doctors to clearly stratify these patients based on their clinical characteristics, the most important of all, to give effective intervention targeting the distinguished risk factors of different hierarchical patients.

Recently, cluster analysis based on clinical features has played an important role in clinical treatment strategies and disease management in many diseases. Ahlqvist E et al. performed a cluster analysis of 8980 patients with adult-onset diabetes based on 6 variables (glutamate decarboxylase antibodies, age at diagnosis, BMI, HbA1c, homoeostatic model assessment 2 estimates of β-cell function, and insulin resistance), and identified 5 replicable clusters (11). They found that different clusters of diabetic patients had significantly different characteristics and risks of diabetes complications, which helps the implementation of precision medicine for diabetes and its complications (11). Meanwhile, they confirmed their results in 3 independent cohorts. Subsequently, more studies reproduced the results of cluster analysis in multiple regions, including China (12, 13). Furthermore, based on this method, some researchers conducted cluster analysis on patients with T2DM, and compared the differences in clinical characteristics, disease progression, treatment response, and complication status of different subgroups, providing a basis for the new strategy for treatment of T2DM (14, 15). Similarly, this method of cluster analysis has also obtained meaningful results in the subgroup classification of diseases such as metabolic-associated fatty liver disease (MAFLD) (16), idiopathic pulmonary fibrosis (IPF) (17), ASCVD (18), bronchiectasis (19), and so on. Furthermore, these results have innovative implications for the early diagnosis of diseases, precise grouping, determining the severity of diseases, and providing optimization of clinical care.

In addition, several studies have explored the potential impact of serum metabolite levels on the development of T2DM and its complications. In 2022, an Updated Systematic Review and Meta-analysis of Prospective Cohort Studies investigated the relationship between metabolomics and the risk of T2DM, the findings revealed significant associations between certain metabolites and the risk of developing type 2 diabetes (20). Recently, according to the study conducted by Wang S et al., there is a positive correlation between a range of amino acids and serum carnitine with T2DM (21). And these studies have identified the related biomarkers, such as branched-chain amino acids, metabolites of phenylalanine, metabolites involved in energy metabolism and lipid metabolism, thereby improved the prevention and treatment of T2DM and its complications (22).

This observational study included the real data of hospitalized patients with chronic complications of T2DM. We aimed to identify characteristic clusters of inpatients with chronic complications of T2DM by K-means clustering based on common clinical variables. We also sought to further analyze the risk of associated chronic complications in each cluster and to compare the differences in metabolite levels among the clusters.

## 2 Materials and methods

### 2.1 Study population

This observational study included patients hospitalized at the Department of Endocrinology, The Second Affiliated Hospital of Dalian Medical University from January 2018 to October 2020. The data were retrieved and collected anonymously through the Yidu Cloud data management platform, mainly including hospitalization measurement indicators, laboratory testing indicators, and diagnostic information on the homepage of discharged medical records. This study was approved by the Ethics Committee of the Second Affiliated Hospital of Dalian Medical University. In this observational study, written informed consent was waived since there was no clinical intervention or treatment involved.

**Figure 1:**
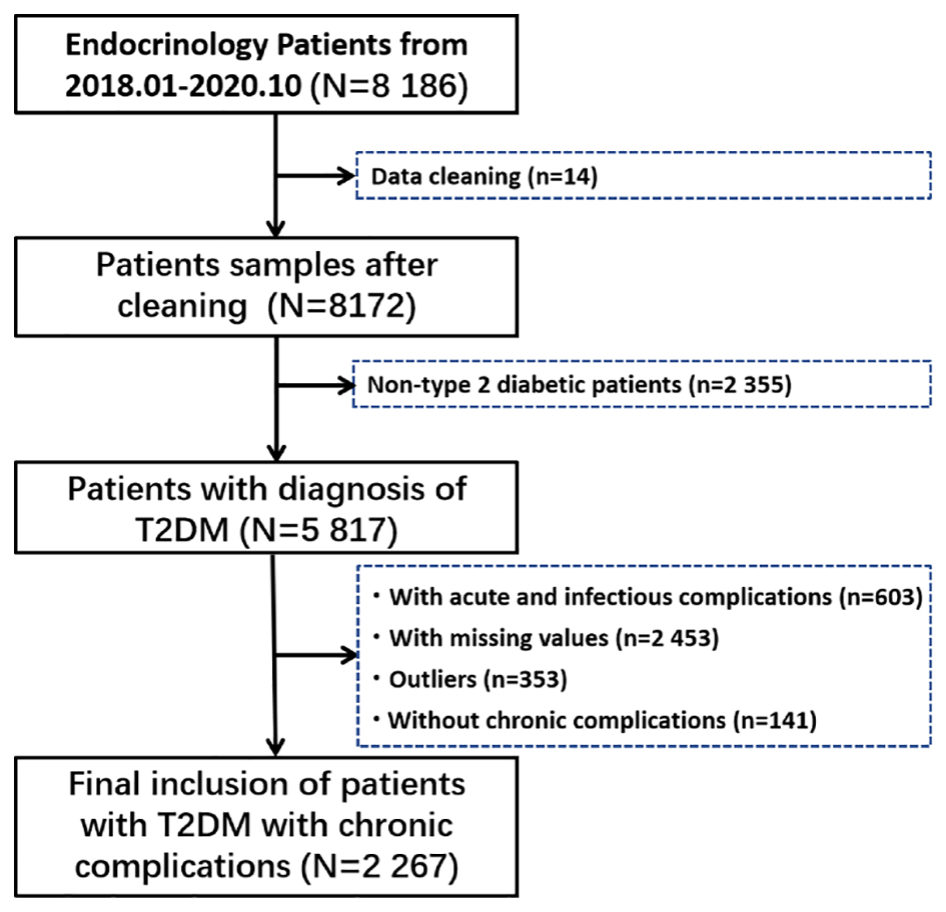
Selecting process for hospitalized patients with chronic complications of T2DM.

A total of 8 186 hospitalized patients were retrieved. First, we carefully checked all the information of 8186 inpatients, and found that there were serious deviations in the information of 14 patients, who were excluded. Then, non-type 2 diabetic patients, patients with acute and infectious complications, patients with incomplete data, patients with outliers (Outliers are defined as five standard deviations above the mean), and patients without chronic complications were gradually excluded, and the specific process is shown in **Figure 1**.

### 2.2 Measurements

The gender and age of hospitalized patients were entered into the medical system based on personal ID cards when registering for admission. Height, Weight, waist circumference (WC), systolic blood pressure (SBP), and diastolic blood pressure (DBP) were measured by professional medical staff after admission. Body mass index (BMI) was calculated by the formula: BMI=weight/height^2^ (Kg/m^2^). All patients were required to fast for at least 8 hours on the first night after admission. The blood samples were collected by professional nurses on the morning of the second day after admission. The inspection items were mainly completed in a special department, and the same items were completed with the same reagents and instruments. The research mainly included liver biochemical indicators[γ-glutamyl transpeptidase (γ-GGT), aspartate transaminase (AST), alanine aminotransferase (ALT)], renal function [Creatinine (Cr), Urea (Ur), uric acid (UA)], fasting blood glucose (Glucose), blood lipid[triglycerides (TG), total cholesterol (TC), low-density lipoprotein-cholesterol (LDL-C), high-density lipoprotein (HDL-C)], fasting insulin, vitamin D (25-(OH)-D), urine creatinine (Cr-urine), urine microalbumin (mAlb-urine) and serum metabolites (see **Supplementary Material 1** for details). Insulin sensitivity index, ISI=1/[fasting blood glucose (mmol/L) × fasting insulin (mU/L)] (23). Homeostatic model assessment (HOMA) insulin resistance, HOMA-IR=[fasting insulin (μU/ml) × fasting blood glucose (mmol/L)]/22.5 (24). Estimated glomerular filtration rate, eGFR=142 × min(Serum creatinine/kappa, 1)^alpha^ × max(Serum creatinine/kappa, 1)^-1.2^ × 0.9938^Age^ × SexFactor (calculation details and variables: For females, the following values are used: SexFactor = 1.012; alpha = -0.241; kappa = 0.7; For males, the following values are used: SexFactor =1; alpha = -0.302; kappa = 0.9) (25).

### 2.3 Definitions

The diagnosis for all inpatients with T2DM was diagnosed by endocrinologists according to the diagnostic criteria proposed by World Health Organization (WHO) Diabetes Expert Committee in 1999 (26). Microangiopathy mainly includes diabetic nephropathy (DN) and diabetic retinopathy (DR). DN was defined as follows: GFR<60 ml/min/1.73 m^2^ and/or urinary albumin to creatinine ratio (UACR) ≥ 30 mg/g after excluding renal diseases caused by other lesions, which has not returned to normal for more than 3 months (27). DR was diagnosed by a professional ophthalmologist after a fundoscopy. Atherosclerotic cardiovascular disease (ASCVD) mainly included atherosclerosis in the aorta, coronary arteries, cerebral arteries, renal arteries, and limb arteries, and were mainly diagnosed by arterial ultrasound, arterial computed tomography angiography (CTA) examination, or coronary angiography, and confirmed by a professional physician. Nervous system complications (NSC) were diagnosed after a professional endocrinologist or professor evaluates the patient’s clinical symptoms and completes the nerve conduction velocity test. Diabetic foot (DF) was diagnosed by clinicians based on clinical symptoms and damage of limb tissues after excluding other lesions.

### 2.4 Cluster analysis

After obtaining the complete data of all hospitalized patients, we selected 11 continuous variables, namely: BMI (kg/m2), SBP (mmHg), DBP (mmHg), Glucose (mmol/L), TG (mmol/L), TC (mmol/L), UA, mAlb, Insulin, ISI and Homa-IR for cluster analysis. The clustering analysis (k-means) is implemented using Python. For the 11 variables screened, min-max normalization was first performed. Then, selecting the optimal number of clusters according to the Silhouette Score (**Supplementary Material 2**). All these variables were the main risk factors for the development of chronic complications of T2DM, and we further named each cluster based on the entirely different characteristics about these variables.

### 2.5 Statistical analysis

Statistical analysis was performed using R Studio, version 4.0.3. Continuous variables conforming to normal distribution were expressed as the mean ± standard deviation (M ± SD) after the normality test, and continuous variables with non-normal distribution were expressed as the median and interquartile range (Median, IQR). Binary categorical variables were expressed as frequencies and percentages. Logistic regression analysis was used to calculate ORs for the occurrence of chronic complications in different sub-clusters. The Kruskal-Wallis H test was used to analyze the differences in metabolite levels of each cluster. The Nemenyi test was used to compare the differences of metabolites in each cluster pairwise. *p*<0.05 was statistically significant.

## 3 Results

### 3.1 Study population and cluster analysis

**Figure 2:**
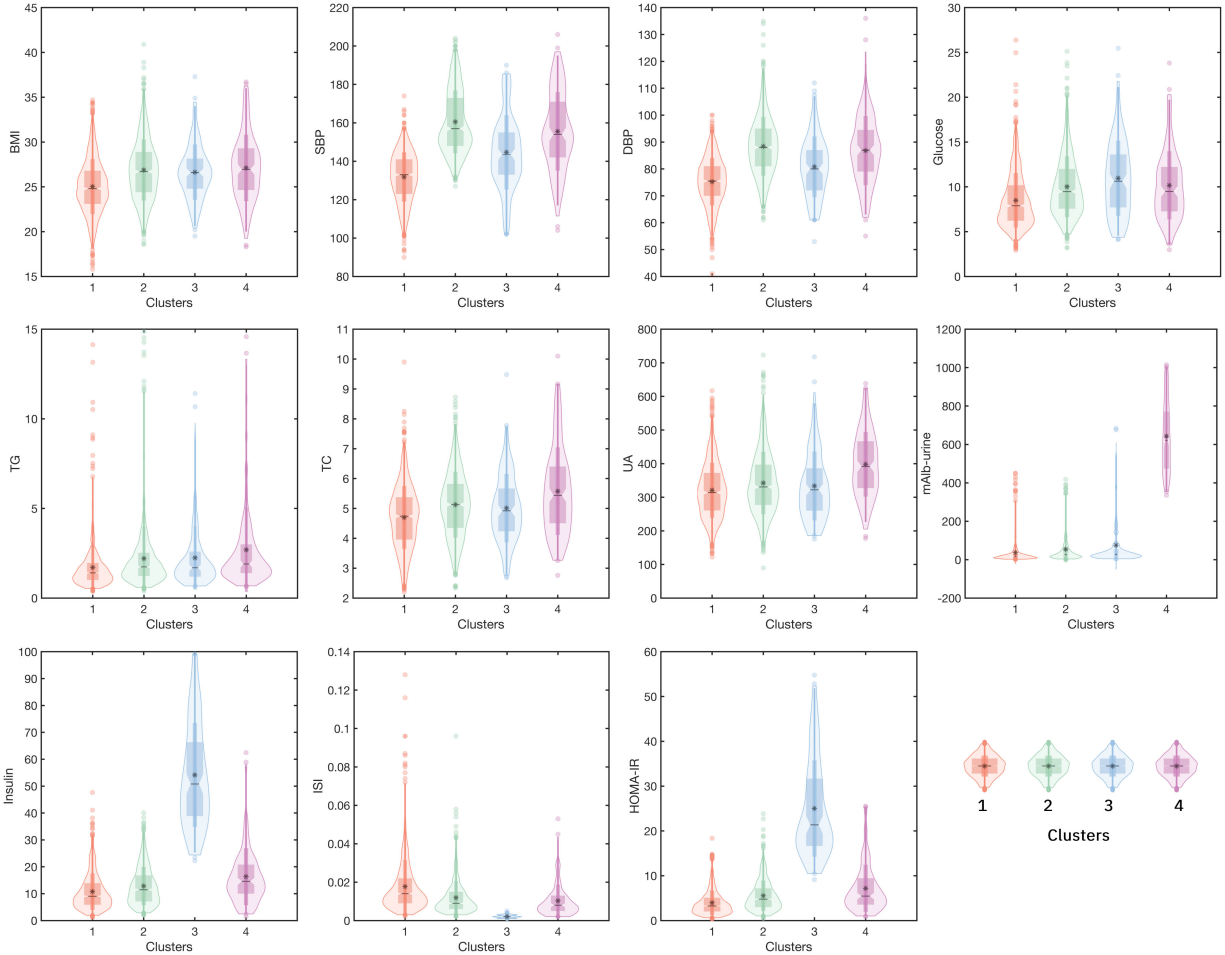
The mean levels of 11 variables in four clusters. (Cluster 1: the Low-Risk cluster; Cluster 2: the Medium-Low-Risk cluster; Cluster 3: the Medium-Risk cluster; Cluster 4: the High-Risk cluster. The horizontal axis represents four different clusters, and the left vertical axis represents the levels of the variable).

In this study, a total of 2 267 hospitalized patients were included for the data analysis, with a median age of 63 years, including 1 230 males (about 54.26%) and 1 037 females (about 45.74%). According to cluster analysis based on 11 clinical variables including BMI, SBP, DBP, Glucose, TG, TC, UA, mAlb (urine), Insulin, ISI, and Homa-IR, all inpatients were divided into four clusters, each cluster has distinct characteristics. The other detailed clustering process and evaluation indicators are in **Supplementary Material 2**. The general situation and clinical status of all inpatients and ones in each cluster are shown in **Table 1**. Cluster 1 was the “Low-Risk”, contained 1112 inpatients (49.05%), characterized by acceptable blood sugar control, good purine, and lipid metabolism (with the lowest glucose, UA, TG, TC, and LDL-C, as well as the highest HDL-C levels among the four clusters), low insulin secretion, high sensitivity, mildest insulin resistance (with the lowest Insulin, the highest ISI, and the lowest Insulin). Cluster 2 was “Medium-Low-Risk”, included 859 inpatients (accounting for 37.89%), characterized by poor blood sugar control (average level<10mmol/L), the highest blood pressure level, and insulin secretion and sensitivity were relatively good, but insulin resistance level was low. Cluster 3 was “Medium-Risk”, included 134 inpatients (accounting for 5.91%), characterized by the worst blood sugar control, the insulin sensitivity was the worst, and the degree of insulin resistance (IR) was also the most serious among the four groups. Cluster 4 was “High-Risk”, included 162 inpatients (accounting for 7.15%), characterized by the highest BMI, the worst lipid metabolism and purine metabolism, and extreme urine microalbumin levels elevated (with the highest TG, TC, LDL-C, UA, and mAlb-urine among four groups). **Figure 2** shows the mean levels of 11 variables in each cluster.

**Table 1:** Basic characteristics of inpatients with chronic complications of T2DM in allocated clusters.

Variables	ALL(N=2267)	Low-Risk (N=1112)	Medium-Low-Risk (N=859)	Medium-Risk (N=134)	High-Risk (N=162)
Gender					
**Female**	1037(45.74%)	501(45.05%)	403(46.92%)	74(55.22%)	59(36.42%)
**Male**	1230(54.26%)	611(54.95%)	456(53.08%)	60(44.78%)	103(63.58%)
**Drink**	534(23.56%)	261(23.40%)	214(24.90%)	22(16.40%)	37(22.80%)
**Smoke**	632(27.88%)	314(28.20%)	235(27.30%)	28(20.80%)	55(33.90%)
**Age**	63(56,70)	63(56,70)	64(56,71)	63(56,70.25)	62(54,70)
**Height**	167(160,173)	167(160,173)	166(160,173)	167(160,172.25)	170(160,176)
**Weight**	71(64,80)	70(62,78)	74(66,83)	75(64.75,80.25)	75(66,89)
**WC**	93(86,100)	91(85,97)	95(88,100)	95(89,100)	97(89,105)
**BMI**	25.70(23.70,28.00)	24.80(23.10,26.80)	26.7(24.40,28.90)	26.6(24.73,28.20)	26.95(24.58,29.30)
**SBP**	144(131,156)	133(123,141)	157(148,173)	143.5(133,155.50)	154(142,172)
**DBP**	80(73,89)	75.5(70,81)	88(81,95)	80(72,87.5)	87(78.75,95.25)
**γ-GGT**	21.79(15.42,33.76)	19.26(14.21,30.07)	23.95(17.08,37.14)	23.16(17.06,34.54)	24.34(17.23,37.83)
**AST**	19.64(16.23,24.3)	19.58(16.06,23.59)	19.83(16.39,25.37)	19.65(16.64,25.29)	19.52(15.98,24.39)
**ALT**	21.45(15.95,30.56)	20.92(15.66,28.75)	22.56(16.56,33.32)	24.66(17.48,32.68)	18.98(14.52,26.66)
**Glucose**	8.61(6.76,11.28)	7.89(6.22,10.18)	9.47(7.55,12.02)	10.625(7.59,13.90)	9.495(7.24,12.24)
**TG**	1.56(1.11,2.28)	1.405(1.01,1.96)	1.74(1.23,2.53)	1.69(1.19,2.60)	1.9(1.39,3.07)
**TC**	4.92(4.12,5.64)	4.74(3.96,5.38)	5.13(4.35,5.82)	4.92(4.23,5.72)	5.44(4.48,6.43)
**LDL-C**	2.59(1.99,3.12)	2.48(1.9,2.98)	2.71(2.08,3.22)	2.61(1.92,3.255)	2.79(2.11,3.54)
**HDL-C**	1.16(0.99,1.37)	1.17(0.99,1.39)	1.16(0.99,1.37)	1.1(0.98,1.32)	1.13(0.94,1.33)
**Crea**	61.34(51.03,73.56)	60.71(50.74,71.69)	60.68(50.83,73.05)	60.75(47.93,73.19)	78.79(60.60,102.58)
**Urea**	5.78(4.91,6.88)	5.63(4.87,6.76)	5.75(4.89,6.80)	5.75(4.86,6.66)	6.905(5.62,8.74)
**eGFR**	100.50(91.89,107.42)	101.05(93.94,107.89)	100.10(92.07,107.41)	101.32(90.86,107.61)	88.47(65.34,105.59)
**UA**	324.44(270.26,388.57)	313.83(260.63,372.38)	330.73(277.05,397.27)	322.15(258.67,392.11)	391.76(325.31,467.37)
**25(OH)D**	17.88(13.25,23.05)	18.41(14.00,23.69)	17.55(12.76,22.55)	17.59(13.08,22.01)	15.64(11.38,21.79)
**Insulin**	10.91(6.77,17.3)	9.04(5.89,13.91)	11.46(7.12,16.81)	50.8(38.66,66.62)	14.60(9.97,21.04)
**ISI**	0.01(0.01,0.01)	0.0136(0.0087,0.0220)	0.0093(0.0062,0.0148)	0.0021(0.0014,0.0027)	0.0081(0.0047,0.0126)
**HOMA-IR**	4.12(2.53,7.01)	3.26(2.02,5.09)	4.77(3.00,7.20)	21.36(16.60,32.05)	5.46(3.53,9.54)
**Cr-urine**	8637.23 (5642.40,12452.00)	8678.75 (5761.21,12850.01)	8653.67 (5622.38,12253.14)	8112.50 (5298.26,11524.25)	8537.11 (4700.38,11887.14)
**mAlb-urine**	22.82(12.24,60.21)	18.125(9.66,32.27)	25.91(14.08,61.38)	27.81(15.41,58.92)	621.42(472.10,772.55)
**Mic**	949(41.86%)	367(33.00%)	373(43.42%)	72(53.73%)	137(84.57%)
**DN**	691(30.48%)	237(21.31%)	272(31.67%)	51(38.06%)	131(80.86%)
**DR**	499(22.01%)	200(17.99%)	188(21.89%)	40(29.85%)	71(43.83%)
**ASCVD**	1859(82.00%)	893(80.31%)	734(85.45%)	95(70.90%)	137(84.57%)
**NSC**	1744(76.93%)	873(78.51%)	641(74.62%)	107(79.85%)	123(75.93%)
**DF**	14(0.62%)	7(0.63%)	3(0.35%)	1(0.75%)	3(1.85%)

### 3.2 Prevalence of chronic complications in each cluster

**Figure 3:**
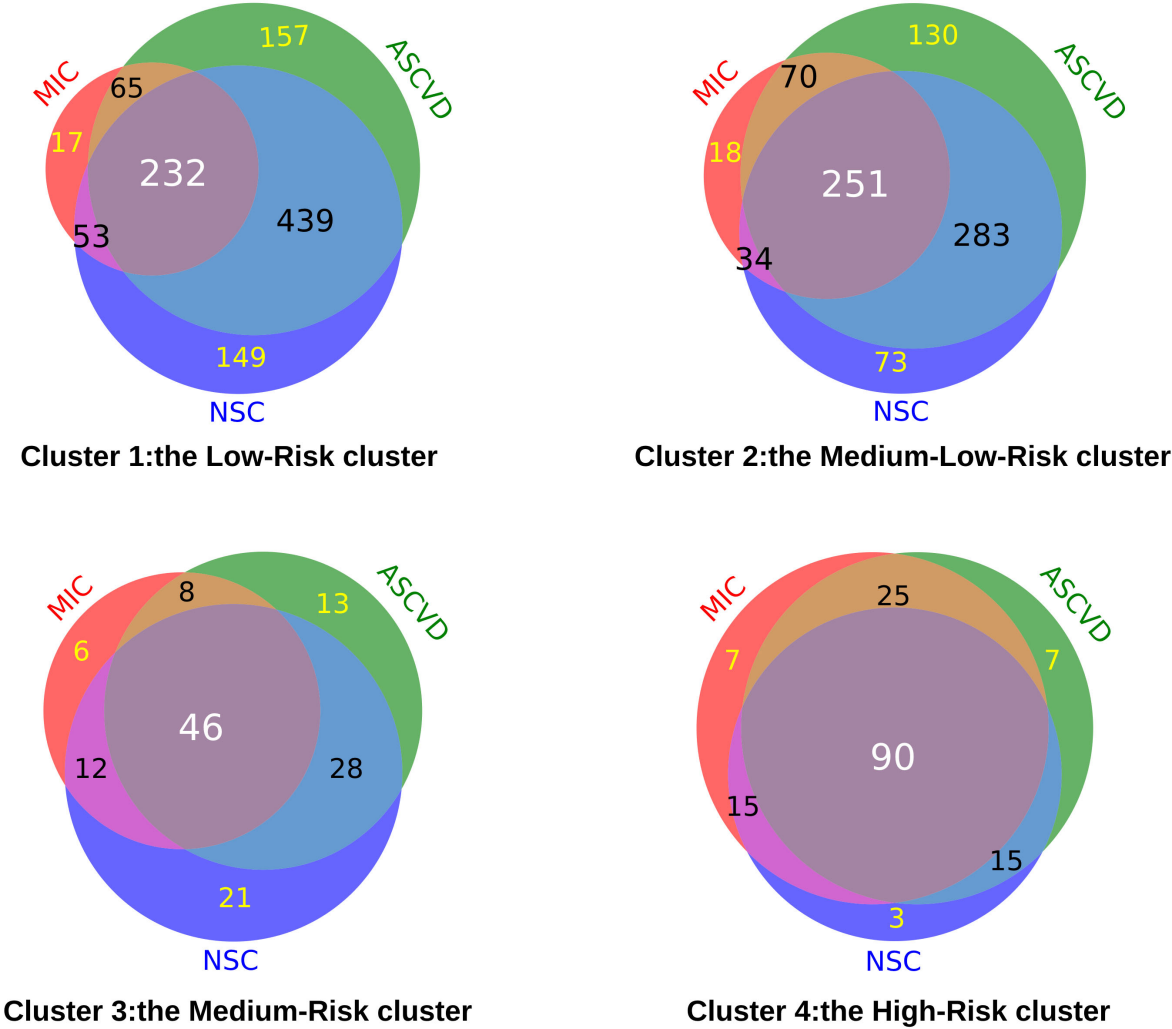
The number of chronic complications of T2DM in each cluster. (MIC, Microangiopathy; ASCVD, Atherosclerotic cardiovascular disease; NSC, Nervous system complications).

Furthermore, to explore the occurrence of chronic complications in each cluster, we counted the incidence of three types of more frequently occurring chronic complications (Microangiopathy, ASCVD, and NSC) in all patients. The proportions of these chronic complications were different in each cluster. For the co-incidence rate of three chronic complications, 232 (20.86%) in the Low-Risk cluster (cluster 1), 251 (29.22%), and 46 (34.33%) in the Medium-Low-Risk cluster (cluster 2) and Medium-Risk cluster (cluster 3) separately. Most seriously, 90 (55.56%) in the High-Risk cluster (cluster 4) had three chronic complications (**Figure 3**).

### 3.3 The risk for chronic complications in each cluster

**Figure 4:**
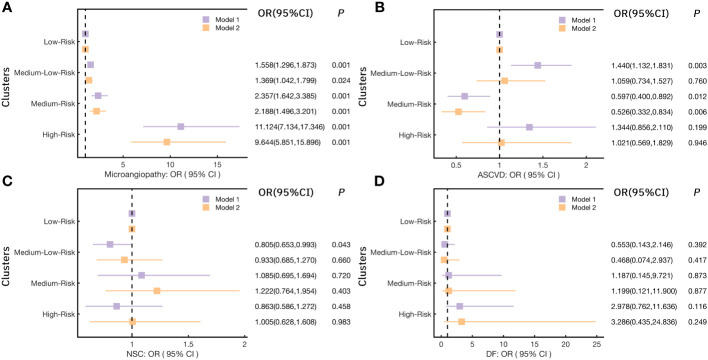
Logistic regression models analyzed the risk for chronic complications in each cluster. **(A)** for Microangiopathy; **(B)** for ASCVD (Atherosclerotic cardiovascular disease); **(C)** for NSC (Nervous system complications); **(D)** for DF (Diabetic foot); (Notes: Model 1: no variables were adjusted; Model 2: adjusted Gender, Drink, Smoke, Age, Weight, WC, BMI, SBP, DBP, TG, TC, LDL-C, HDL-C, γ-GGT, AST, ALT, eGFR, UA, Crea, 25(OH)D).

Here we further illustrated the risk trends for various chronic complications in each cluster by logistic regression analysis (**Figure 4**). As shown in **Figure 4A**, in model 1, the risk of microangiopathy in the Medium-Low-Risk cluster (cluster 2), the Medium-Risk cluster (cluster 3), and the High-Risk cluster (cluster 4) was higher than the Low-Risk cluster (cluster 1) (ORs>1, *p*<0.05). Significant high trends were also observed for all three clusters after 20 variables (Gender, Drink, Smoke, Age, WC, BMI, SBP, DBP, TG, TC, LDL-C, HDL-C, γ-GGT, AST, ALT, eGFR, UA, Crea, 25(OH)D) were adjusted in model 2. For the risk of ASCVD, the Medium-Low-Risk (Cluster 2) was associated with high risk compared with the Low-Risk cluster (cluster 1) [*OR(95%CI)*:1.440(1.132,1.831), *p*=0.003]. However, after adjusting for potential confounders, this association was relatively low [*OR(95%CI)*:1.059(0.734,1.527), *p*=0.76]. It is worth mentioning that the Medium-Risk cluster (cluster 3) has a significant reduction of the ASCVD risk [*OR(95%CI)*:0.597(0.400,0.892), *p*=0.012] (**Figure 4B**). Similarly, this result was observed in model 2 [*OR(95%CI)*: 0.526 (0.332,0.834), *p*=0.006]. Compared with the Low-Risk cluster (cluster 1), a significant reduction of the NSC risk was observed only in the Medium-Low-Risk (Cluster 2) in model 1[*OR(95%CI)*:0.805(0.653,0.993), *p*=0.043] (**Figure 4C**). In addition, no significant difference was observed in all models for the risk of DF (**Figure 4D**). More detailed results are shown in **Supplementary Table 3-1**.

**Figure 5:**
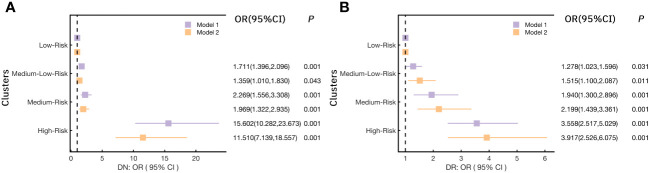
Logistic regression models analyzed the association of **(A)** DN (diabetic nephropathy) and **(B)** DR (diabetic retinopathy) with each cluster.(Notes: Model 1: no variables were adjusted; Model 2: adjusted Gender, Drink, Smoke, Age, Weight, WC, BMI, SBP, DBP, TG, TC, LDL-C, HDL-C, γ-GGT, AST, ALT, eGFR, UA, Crea, 25(OH)D).

Considering the significant high risk of microangiopathy was observed in the other three clusters as compared to the Low-Risk cluster. In addition, we further explored the risk for DN and DR (the two most elements in microangiopathy) in each cluster (**Figure 5**) and **Supplementary Table 3-2**. When Compared with the Low-Risk cluster (cluster 1), the *OR(95%CI)* of DN in the Medium-Low-Risk cluster (cluster 2), the Medium-Risk cluster (cluster 3), and the High-Risk cluster (cluster 4) were: 1.711(1.396,2.096), 2.269(1.556,3.308), and 15.602(10.282,23.673) (all *p*<0.05), respectively. The associations remained statistically significant after further adjusting in model 2 (all *p*<0.05) (**Figure 5A**). We also noted that the Medium-Low-Risk cluster (cluster 2), the Medium-Risk cluster (cluster 3), and the High-Risk cluster (cluster 4) had more DR risk, the *OR(95%CI)* were 1.278(1.023,1.596), 1.940(1.300,2.896), and 3.558(2.517,5.029) (all *p*<0.05). These associations were consistent after multivariable adjustment in model 2 (**Figure 5B**).

### 3.4 Comparison of metabolite profile in each cluster

Previous research has demonstrated that alterations in metabolites and metabolic pathways play a significant role in the onset of diabetes and its associated complications (20). Here, in order to investigate the variations of metabolites in distinct clusters, the Kruskal-Wallis H test was analyzed. The results showed 24 metabolites with statistically significant distribution differences among the clusters (**Table 2**).

**Table 2:** The specific differences of metabolites among 4 clusters.

Variables	Low-Risk	Medium-Low-Risk	Medium-Risk	High-Risk	*P*
**Ala**	184.625(141.138,225.678)	188.670(147.960,229.910)	181.600(154.918,238.960)	176.705(133.135,218.523)	0.023
**Arg**	3.265(2.073,4.738)	2.940(1.920,4.590)	2.785(1.645,4.145)	3.365(2.240,5.368)	0.001
**Asp**	27.425(19.725,35.898)	25.670(18.430,33.970)	26.515(18.850,34.685)	27.785(21.183,35.863)	0.014
**Cit**	21.755(16.493,27.810)	21.810(16.930,27.160)	22.225(17.393,28.113)	24.045(18.315,30.448)	0.006
**Cys**	295.970(266.348,330.780)	312.130(280.990,346.030)	304.175(263.718,340.828)	321.265(290.273,370.673)	<0.001
**Met**	14.990(12.460,17.650)	14.670(12.460,17.170)	15.070(13.128,18.255)	13.950(11.728,16.563)	0.015
**Phe**	14.155(10.470,19.885)	13.300(9.730,18.340)	13.480(10.113,18.290)	14.130(10.520,19.195)	0.014
**Pip**	202.395(140.013,275.530)	182.390(129.910,244.420)	178.515(132.613,222.075)	183.755(128.178,245.068)	<0.001
**Thr**	25.915(20.733,32.350)	24.510(19.740,30.680)	24.160(20.178,28.895)	23.825(19.615,29.700)	<0.001
**Tyr**	50.785(39.688,62.740)	50.440(40.560,62.360)	52.360(41.103,65.030)	42.980(34.880,54.573)	<0.001
**Val**	145.335(123.813,169.685)	149.770(127.520,175.270)	155.865(129.628,178.603)	143.850(119.735,168.465)	0.002
**C2**	11.740(8.893,15.018)	11.660(9.230,14.730)	11.160(8.008,14.600)	12.740(10.023,15.818)	0.035
**C3**	1.470(1.090,1.968)	1.540(1.140,2.070)	1.630(1.158,2.040)	1.605(1.093,2.243)	0.024
**C4**	0.180(0.130,0.230)	0.180(0.140,0.240)	0.170(0.130,0.243)	0.200(0.160,0.280)	<0.001
**C4:0-OH**	0.050(0.040,0.070)	0.050(0.040,0.070)	0.050(0.040,0.080)	0.060(0.040,0.090)	0.011
**C5**	0.110(0.080,0.140)	0.110(0.090,0.150)	0.110(0.090,0.150)	0.130(0.090,0.160)	0.001
**C5DC**	0.070(0.050,0.100)	0.070(0.040,0.110)	0.065(0.040,0.100)	0.090(0.050,0.120)	0.003
**C6**	0.070(0.050,0.090)	0.070(0.060,0.100)	0.070(0.050,0.090)	0.080(0.060,0.100)	0.001
**C6DC**	0.340(0.210,0.510)	0.350(0.230,0.530)	0.330(0.220,0.533)	0.400(0.260,0.580)	0.022
**C5OH**	0.200(0.150,0.270)	0.220(0.160,0.290)	0.230(0.160,0.313)	0.230(0.170,0.310)	<0.001
**C14**	0.060(0.040,0.090)	0.070(0.050,0.090)	0.060(0.040,0.080)	0.070(0.050,0.100)	0.011
**C16**	0.830(0.660,1.050)	0.880(0.680,1.110)	0.870(0.760,1.113)	0.930(0.730,1.173)	<0.001
**C18**	0.490(0.380,0.620)	0.490(0.380,0.600)	0.550(0.415,0.653)	0.545(0.408,0.653)	0.003
**C26**	0.030(0.020,0.040)	0.030(0.020,0.040)	0.030(0.020,0.040)	0.030(0.020,0.040)	0.037

Ala, Alanine; Arg, Arginine; Asp, Aspartate; Cit, Citrulline; Cys, Cysteine; Met, Methionine; Phe, Phenylalanine; Pip, Piperamide; Thr, Threonine; Tyr, Tyrosine; Val, Valine; C2:Acetyl-carnitine; C3:Propionyl-carnitine; C4:Butyryl-carnitine; C4:0-OH, Hydroxybutyryl-carnitine; C5:Isovaleryl-carnitine; C5DC, Glutaryl-carnitine; C6:Hexanoyl-carnitine; C6DC, Adipyl carnitine; C5OH, Hydroxyisovaleryl-carnitine; C14:Myristoyl-carnitine; C16:Palmitoyl-carnitine; C18:Octadecanoyl-carnitine; C26:26-carboacyl carnitine.

**Figure 6:**
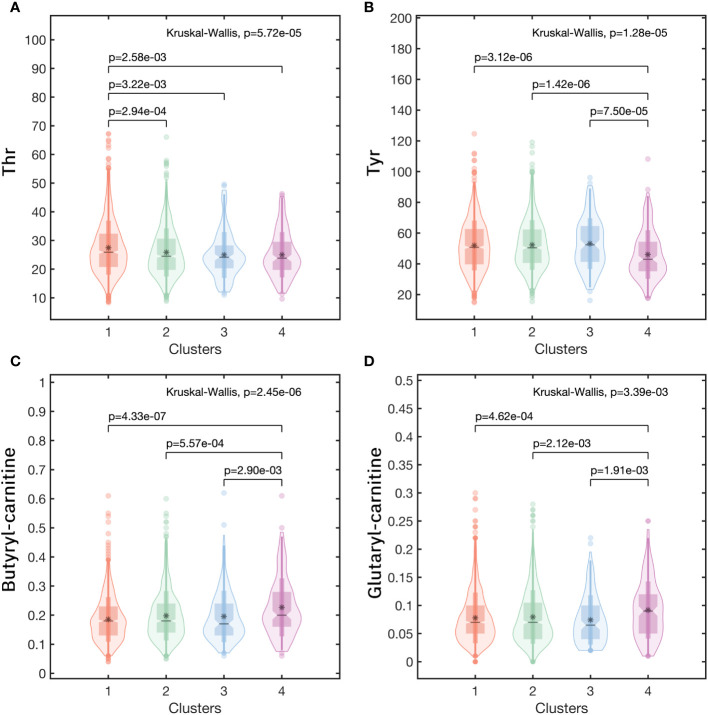
The metabolic profiles in clusters of **(A)** Thr, **(B)** Tyr, **(C)** Butyryl carnitine, **(D)** Glutaryl carnitine. (Note:Cluster 1: the Low-Risk cluster; Cluster 2: the Medium-Low-Risk cluster; Cluster 3: the Medium-Risk cluster; Cluster 4: the High-Risk cluster).

To further clarified the specific metabolites in every cluster, we used each cluster as the reference and compared the other clusters with this one. For the Low-Risk cluster (cluster 1), only one metabolite had a significant difference with the other three clusters, that was Thr, significantly high in cluster 1 (**Figure 6A**). For the High-Risk cluster (cluster 4), Glutaryl carnitine and Butylcarnitine were higher and Tyr was lower than the other three clusters (all *p*<0.05) (**Figures 6B-D**). However, for the Medium-Low-Risk cluster (Cluster 2) and the Medium-Risk cluster (Cluster 3), there was no metabolites that had a significant difference compared with the other three clusters.

## 4 Discussion

The K-means method is a traditional and relatively simple machine learning method (28). It had been used for iterative subspace projection and clustering, consensus clustering (29), disease phenotype recognition (11, 16), and the results hold significant value in the clinical progression study of the disease. In this retrospective real data study, 4 clusters of patients with chronic complications of T2DM were divided using K-means cluster analysis based on 11 common clinical variables. Each cluster showed substantially different phenotypes in clinical patterns and the risks of microangiopathy, ASCVD, and NSC were significantly differential enriched in each cluster. In the current analysis, we also found distinct metabolic characteristics and specific metabolites in each cluster.

Cluster analysis based on clinical features is of great value in disease subtype decision-making. Several published studies in recent years have tried to phenotype T2DM patients. These studies proved novel hierarchical clusters of T2DM patients could express significant differences including clinical characteristics, disease progression, complications, and treatment responses (14, 15, 30). To the best of our knowledge, limited studies focused on T2DM patients with chronic complications. In this current study, we stratified T2DM patients with chronic complications into 4 distinct clusters based on clinical data: the Low-Risk cluster, the Medium-Low-Risk cluster, the Medium-Risk cluster, and the High-Risk cluster. The four clusters showed different risks of the accumulation of chronic complications for T2DM patients, the High-Risk cluster had 55.55% of the patients with comorbidity of three chronic complications. Moreover, patients in the High-Risk cluster had more risk to have microangiopathy (OR:9.644, *p*<0.001, after adjusting for 20 covariates), especially for DR (OR:15.602, *p*<0.001, after adjusting for 20 covariates). That means patients in the High-Risk cluster need to pay more attention to preventing the occurrence of multiple complications of T2DM.

What is special is that, compared with the Low-Risk cluster, the ASCVD risk of the Middle-Low-Risk cluster was higher (OR:1.440, *p*=0.003, no variables were adjusted), and it was also relatively high in the High-Risk cluster (OR >1, although *p*<0.05, regardless of adjustment for covariates), but the Medium-Risk cluster shows a lower ASCVD risk (OR:0.526, *p*=0.006, after adjusting for 20 covariates). Tracing back to the variable characteristics of each cluster in **Table 2**, it was found that the corresponding levels of SBP and DBP in the Middle-Low-Risk cluster and High-Risk cluster were higher, and also showed relatively higher ASCVD risk values; while the blood pressure control level of patients in the Middle-Risk cluster was slightly better than the former two clusters, meanwhile, the middle-risk group showed a lower risk of ASCVD, regardless of model 1 or model 2, with a *p*-value of less than 0.05. This result reflects that the risk of ASCVD in patients may be closely related to blood pressure levels. Previous studies have also suggested that blood pressure is one of the risk factors for ASCVD in patients with T2DM (31), and a meta-analysis also found that antihypertensive treatment reduces the risk of CVD events in patients with T2DM (32). *Stefano Ciardullo* et al. also found that the seasonal change of SBP is the main factor leading to the seasonal variation of ASCVD risk score in patients with T2DM (33).

It was also found that patients in the Medium-Low-Risk cluster had a lower risk of developing NSC (OR=0.805, *p*=0.043), but this trend lost statistical significance after adjusting for 20 covariates. This may be related to the joint effect of multiple NSC risk factors in the pathogenic process. *Xiuxiu Liu* et al. pointed out that the risk factors for diabetic peripheral neuropathy (DPN) are: the duration of diabetes, age, glycosylated hemoglobin A1c(HbA1c), and DR, but BMI, smoking, total triglyceride (TG), and total cholesterol (TC) did not increase the risk of DPN (34). The study on the risk factors of DPN in young patients with diabetes found that risk factors for DPN in youth with type 1 diabetes were older age, longer diabetes duration, smoking, increased DBP, obesity, increased LDL-C and triglycerides (TG), and lower HDL-C. While in youth with T2DM, risk factors were older age, male sex, longer diabetes duration, smoking, and lower HDL-C (35). In addition, an observational study of childhood and adolescents with diabetes found that people with T2DM were more likely to develop DPN than those with type 1 diabetes (36). Overall, the pathogenesis and core pathogenic factors of neurological complications of type 2 diabetes still need to be further studied.

In the metabolite analysis of the 4 clusters, we found that 24 metabolites had statistically significant differences among the clusters, and screened out metabolites with typical characteristics in some clusters. Glutaryl carnitine and Butylcarnitine increased while Tyr decreased in the High-Risk cluster, and Thr increased in the Low-Risk cluster. These findings will provide new possibilities for risk intervention targeting metabolite levels in patients with chronic complications of T2DM. These characteristic metabolites can be used as biomarkers for the screening, diagnosis, and prediction of chronic complications of T2DM, which will help to understand the metabolic information and metabolic pathways of chronic complications of T2DM and explore the possible mechanisms of complications, thus providing possible targets for its treatment (22). For example, *J Ricardo Lucio-Gutiérrez* et al. used partial least squares analysis (PLS-DA) to obtain the PLS-DA model for distinguishing type 2 diabetic nephropathy based on urinary metabolites, and it was well validated (37). This provides a new diagnostic option for the diagnosis of asymptomatic DN in patients with T2DM.

In the correlation analysis of characteristic metabolites and chronic complications, we found that Thr was negatively correlated with microangiopathy(especially DN), but positively correlated with ASCVD. And Tyr was negatively correlated with microangiopathy (regardless of DN or DR) and NSC. Both Butylcarnitine and Glutaryl carnitine were positively associated with ASCVD risk. *Cornelia G Bala* et al. found that the disruption of the tyrosine biosynthesis pathway might be disrupted related to the high oxidative stress response in patients with type 2 diabetes (38). Oxidative stress is a key pathogenic factor in diabetic complications and can lead to the development of microangiopathy and ASCVD (39, 40). Changes in metabolite levels may reflect the occurrence of multiple pathogenic mechanisms. Therefore, further research on the underlying mechanisms involved in changes in other metabolites is particularly important for the study of disease progression. And thereby reducing the huge disease burden of diabetes (41).

Our study is based on real data of hospitalized patients without any missing values, which can better reflect the practical situation of patients with chronic complications of T2DM. With a relatively ideal amount of data and a balanced male-to-female ratio, the cluster analysis obtained the cluster characteristics of patients with a high risk of chronic complications of T2DM. The relationship between metabolic changes and chronic complications was analyzed from the level of metabolites, which provides new ideas and targets for the prevention and treatment of chronic complications of T2DM and is helpful for the precise treatment of chronic complications of T2DM. However, our study also has certain limitations. It is a single-center study, which only includes patients from Dalian, Northeast China, and has not yet included subjects from other countries and regions. At the same time, this study was also retrospective and failed to observe the progression of chronic complications in each subcluster. In order to extend our research and comprehensively promote the guiding significance of our research results to clinical decision-making, it is very necessary to carry out corresponding prospective follow-up research and observe the treatment response. In the future, we will continue to improve this part of the research to enhance its value of the research.

## 5 Conclusions

The complication status of patients with chronic complications of T2DM has obvious cluster characteristics, and the performance of different target organ damage risks is not completely consistent. However, the current classification of complications has not considered the impact of these pathogenic risk factors on the occurrence of complications. The clustering results of this study reflect the clustering characteristics of different risk levels of target organ damage and the clustering characteristics of multiple complications. Different clusters also had significant differences in the levels of most metabolites, which helps to explore the potential role of the pathogenic mechanism of metabolic pathway alterations in the progression of complications. This will provide novel decision support for the prevention and treatment of chronic complications of T2DM.

## Data availability statement

The raw data supporting the conclusions of this article will be made available by the authors, without undue reservation.

## Ethics statement

The studies involving humans were approved by the Ethics Committee of the Second Affiliated Hospital of Dalian Medical University. The studies were conducted in accordance with the local legislation and institutional requirements. Written informed consent for participation was not required from the participants or the participants’ legal guardians/next of kin in accordance with the national legislation and institutional requirements.

## Author contributions

CW, YL, KD and CL performed data analysis, interpretation and manuscript writing. CW, HZ and JW supervised the data collection and research collaboration. CW, CL, GW, HZ and XL participated in data collection and literature search. HZ, CW and XL designed the experiment and supervised the overall progress. All authors contributed to the article and approved the submitted version.
