# Supplementary material for: Unsupervised cluster analysis of clinical and metabolite characteristics in patients with chronic complications of T2DM: an observational study of real data
Source: Front Endocrinol (Lausanne). 2023 Oct 20;14:1230921. doi: 10.3389/fendo.2023.1230921 (PMC10623421; doi:10.3389/fendo.2023.1230921)
Supplement: Supplementary file 1 [file DataSheet_1.pdf]

Supplementary Material

Unsupervised cluster analysis of clinical and metabolite characteristics in patients with chronic complications of T2DM: an observational study of real data

Cuicui Wang[12†], Yan Li[3†], Jun Wang[2†], Kunjie Dong[4], Chenxiang Li[4], Guiyan Wang[5], Xiaohui Lin[4*], Hui Zhao[1*]

***Correspondence:**
Xiaohui Lin**:**datas@dlut.edu.cn
Hui Zhao**:**zhaohui@dmu.edu.cn

[†]These authors contributed equally to this wor

1 Supplementary Material 1

Metabolite measurements will be performed immediately after blood samples from all patients are collected (tests are typically completed and reported within 48 hours). Metabolites were measured by LC-MS/MS(liquid chromatography-tandem mass spectrometry) at the Dalian Institute of Chemical Physics, Chinese Academy of Sciences. LC-MS/MS was performed on an API 3200 quadrupole mass spectrometer (Applied Biosystem, Waltham, MA, USA) equipped with an electrospray ionization (ESI) probe, Chemo View 1.4.2, and Agilent 1200 high-performance liquid chromatography (Agilent Technologies, Santa Clara, CA, USA). Metabolites detected include 22 amino acids and 25 carnitines. Details as follows:

1. 22 amino acids:

Alanine(Ala), Arginine(Arg), Asparagine(Asn), Aspartate(Asp), Citrulline(Cit), Cysteine(Cys), Glutamine(Gln), Glutamic acid(Glu), Glycine(Gly), Homocysteine (HCY), Histidine(His), lysine(Lys), Methionine(Met), Phenylalanine(Phe), Ornithine(Orn), Piperamide, Proline(Pro), Serine(Ser), Threonine(Thr), Tryptophan (Trp), Tyrosine(Tyr), Valine(Val).

2. 25 carnitines:

Acetyl-carnitine(C2),Propionyl-carnitine(C3),Butyryl-carnitine(C4),Butyldiacyl-carnitine(C4DC),Hydroxybutyryl-carnitine(C4:0-OH),Isovaleryl-carnitine(C5),Glutaryl-

carnitine(C5DC),Penenyl-carnitine(C5:1),Hexanoyl-carnitine(C6),Decanoyl-carnitine(C10),Adipyl -carnitine(C6DC),Hydroxyisovaleryl-carnitine(C5OH),Lauroyl-carnitine(C12),Hydroxymyristoyl-carnitine(C14OH), Myristoyl-carnitine(C14), Myristodiacyl-carnitine(C14DC), Myristomyl-carnitine(C14:1),Palmitoyl-carnitine(C16),Hydroxypalmitoyl-carnitine(C16:OH),Hydroxypalmitoyl-carnitine(C16:1OH),Octadecanoyl-carnitine(C18),Eicosyl-carnitine(C20),Docoacyl-carnitine(C22),24-carboacyl-carnitine(C24),26-carboacyl-carnitine(C26).

2 Supplementary Material 2

In the study, the cluster analysis of all patients with chronic complications of T2DM adopts the K-means clustering method, the number of clusters and corresponding evaluation indicators are as follows.

2.1 Supplementary Figures

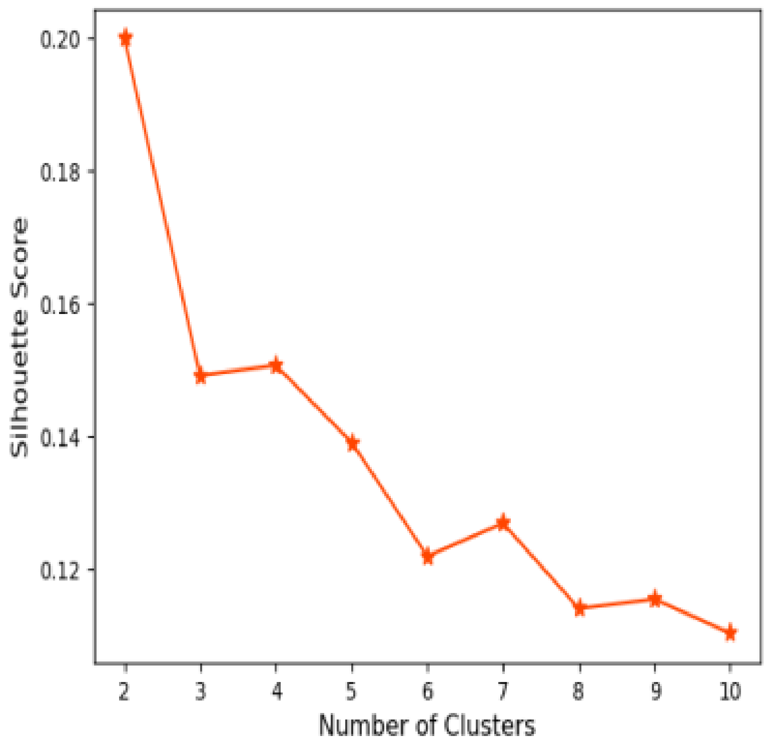

Supplementary Figure 1. The evaluation index for k-means cluster analysis.Silhouette Score-middle picture.

2.2 Supplementary Tables

Supplementary Table 1 | The evaluation indexes of K-means cluster analysis.

K	Silhouette Score
**K=2**	0.199945296
**K=3**	0.149122703
**K=4**	0.150674789
**K=5**	0.139072262
**K=6**	0.121915976
**K=7**	0.126870335
**K=8**	0.114030605

3 Supplementary Material 3

Supplementary Table 2 | The differences in the distribution of metabolites among the four subclusters were analyzed by the Kruskal-Wallis H test.

Variables	K	df	*p*
**Ala**	**9.579**	**3**	**0.023**
**Arg**	**16.998**	**3**	**0.001**
**Asn**	4.226	3	0.238
**Asp**	**10.692**	**3**	**0.014**
**Cit**	**12.289**	**3**	**0.006**
**Cys**	**72.921**	**3**	**＜0.001**
**Gln**	5.027	3	0.17

**Glu**	2.49	3	0.477
**Gly**	2.823	3	0.42
**HCY**	4.935	3	0.177
**His**	4.801	3	0.187
**Lys**	3.972	3	0.265
**Met**	**10.519**	**3**	**0.015**
**Phe**	**10.557**	**3**	**0.014**
**Orn**	5.679	3	0.128
**Pip**	**23.345**	**3**	**＜0.001**
**Pro**	1.549	3	0.671
**Ser**	1.445	3	0.695
**Thr**	**22.273**	**3**	**＜0.001**
**Try**	5.586	3	0.134
**Tyr**	**25.392**	**3**	**＜0.001**
**Val**	**14.43**	**3**	**0.002**
**C2**	**8.632**	**3**	**0.035**
**C3**	**9.482**	**3**	**0.024**
**C4**	**28.815**	**3**	**＜0.001**
**C4DC**	5.889	3	0.117
**C4:0-OH**	**11.148**	**3**	**0.011**

**C5**	**15.855**	**3**	**0.001**
**C5DC**	**13.667**	**3**	**0.003**
**C5:1**	5.289	3	0.152
**C6**	**17.203**	**3**	**0.001**
**C10**	3.056	3	0.383
**C6DC**	**9.585**	**3**	**0.022**
**C5OH**	**17.985**	**3**	**＜0.001**
**C12**	4.096	3	0.251
**C14OH**	2.342	3	0.505
**C14**	5.392	3	0.145
**C14DC**	1.411	3	0.703
**C14:1**	**11.124**	**3**	**0.011**
**C16**	**20.208**	**3**	**＜0.001**
**C16OH**	4.678	3	0.197
**C16:1OH**	2.198	3	0.532
**C18**	**14.126**	**3**	**0.003**
**C20**	7.727	3	0.052
**C22**	4.112	3	0.25
**C24**	4.117	3	0.249
**C26**	**8.491**	**3**	**0.037**
